# Low E-cadherin expression is associated with poor prognosis in pulmonal adenocarcinoma

**DOI:** 10.1038/s41598-026-45409-0

**Published:** 2026-03-30

**Authors:** Fiete Gehrisch, Kiara A. Schmid, Martina Kluth, Georgia Makrypidi-Fraune, Katharina Möller, Maximilian Lennartz, Veit Bertram, Florian Lutz, Stefan Steurer, Philipp Busch, Birgit Hantzsch-Kuhn, Martin Reck, Till Olchers, David Benjamin Ellebrecht, Christoph Fraune, Ronald Simon, Guido Sauter, Sönke von Weihe

**Affiliations:** 1 Institute of Pathology, University Medical Center Hamburg-Eppendorf, Martinistr. 52, 20246 Hamburg, Germany; 2 Department of General, Visceral and Thoracic Surgery, University Medical Center Hamburg-Eppendorf, Hamburg, Germany; 3 Department of Thoracic Oncology, Airway Research Center North, German Center for Lung Research, LungenClinic, Grosshansdorf, Germany; 4 Department of Thoracic Surgery, Airway Research Center North, German Center for Lung Research, LungenClinic, Grosshansdorf, Germany; 5 Institute of Pathology, Clinical Center Osnabrueck, Osnabrueck, Germany

**Keywords:** Biomarkers, Cancer, Oncology

## Abstract

E-cadherin is a critical cell adhesion molecule with a tumor suppressive role. Reduced membranous E-cadherin expression is considered a central step in the progression and metastatic spread in human cancers. To study the prevalence and potential role of reduced E-cadherin expression in different lung cancer subtypes, E-cadherin expression was analyzed in more than 850 resected lung cancers by IHC in a tissue microarray format. A membranous E-cadherin staining was seen in 779 (96.9%) of the 804 interpretable tumor samples. A loss of E-cadherin expression was slightly more frequent in adenocarcinomas (ACs; 1.1%) than in squamous cell carcinomas (SCCs; 0.0%; p < 0.0001). Among the less common tumor entities, E-cadherin immunostaining was absent in 57.1% of 35 mesotheliomas, but retained in all cases of carcinoid (n = 52), large cell neuroendocrine carcinoma (n = 19), undifferentiated large cell carcinoma (n = 3), carcinosarcoma (n = 2), pleiomorphic carcinoma (n = 11), adenosquamous carcinoma (n = 8), mucoepidermoid carcinoma (n = 1), lymphoepithelial carcinoma (n = 1), and SMARCA4-deficient undifferentiated tumors (n = 1). Reduced E-cadherin immunostaining was significantly linked to advanced pT stage (p = 0.0265) and high grade (p = 0.0351) in ACs. Univariate outcome analysis revealed a significant association between E-cadherin expression and patient survival in all NSCLCs (p < 0.0001) and in ACs (p = 0.0133). It is concluded that loss of E-cadherin expression occurs in only a small subset of NSCLCs, that it is more frequent in ACs compared to SCCs and that reduced E-cadherin expression is linked to poor prognosis and unfavorable histopathological features in ACs. Should targeted therapy become available in the future, a small subset of pulmonary ACs and many mesotheliomas could be candidates for treatment.

**Supplementary Information:**

The online version contains supplementary material available at 10.1038/s41598-026-45409-0.

## Introduction

Lung cancer is the leading cause for cancer-related deaths worldwide, accounting for an estimated 125,000 deaths annually in the U.S. alone^1^. During their lifetime 1 in 17 women and 1 in 16 men in the U.S. will be diagnosed with this disease^1^. Lung cancer has traditionally been classified into two histological subtypes: small-cell lung cancer and non-small-cell lung cancer (NSCLC), with NSCLC making up for 85% of all cases^2^. Among NSCLC, adenocarcinoma (AC) (50%) and squamous cell carcinoma (SCC) (20–30%) are the most prevalent subtypes^3,4^. Despite therapeutic advances, the 5-year survival rate remains at approximately 20%^1^, reflecting that NSCLC remains an incurable disease for the majority of patients^5^. This persistent challenge highlights the urgent need to further develop therapeutic strategies and to identify and validate reliable prognostic and predictive biomarkers capable of stratifying patients who may most benefit from aggressive treatment approaches^6^.

E-cadherin is a critical cell adhesion molecule with a tumor suppressive role. It is expressed in nearly all epithelial tissues and is continuously regenerated, with a half-life of 5–10 hours^7^. Reduced E-cadherin expression is considered a central step in the progression and metastatic cascade in human cancers^8–10^ as it impairs binding between cells. As a consequence, contact inhibition of proliferation is suppressed, cells reach confluence^10^ and evolve epithelial to mesenchymal transition (EMT)^11^. Data from several studies suggested a link between reduced E-cadherin expression and advanced clinicopathological features, metastatic disease, and poor prognosis in patients with NSCLC^12–28^, as well as in separate cohorts of ACs^24,29–31^ and SCCs^22,31–33^. However, these findings remain methodologically heterogeneous, confined to rather small patient cohorts and reported expression levels vary considerably across studies. Additionally, other studies have not found associations between reduced E-cadherin expression and unfavorable clinicopathological features or poor prognosis^15,22,24,26,27,33–39^, or have even reported opposite results^40^. Lastly, data on expression levels and clinical significance of E-cadherin in less frequent subtypes of lung cancer remain sparse.

To further elucidate the distribution and clinical relevance of E-cadherin expression in human lung cancer, we took advantage of a preexisting tissue microarray (TMA) containing 857 lung cancer tissue samples from 12 subtypes and conducted a comprehensive analysis by immunohistochemistry (IHC) using a previously validated assay^41^.

## Materials and methods

### Tissue microarrays (TMAs)

Our TMA cohort consisted of one sample each from 858 surgically resected lung cancer cases. All specimens originated from the archives of the Institute of Pathology, University Medical Center Hamburg, Germany. According to local regulations (HmbKHG, §12) the responsible IRB (Ethics commission Hamburg) (reference number: WF-049/09) approved the use of archived remnants of diagnostic tissues for manufacturing of TMAs and their analysis for research purposes as well as patient data analysis and waived the need for consent to participate in this study. All work has been carried out in compliance with the Helsinki Declaration. Histopathological data available for these samples included tumor stage (pT), grade, lymph node status (pN), and resection margin (R), as summarized in Table 1. Follow-up data regarding overall survival (OS) was available for 728 patients, comprising 400 cases of adenocarcinoma (AC) and 208 cases of squamous cell carcinoma (SCC). The TMA manufacturing process has previously been described in detail^42–44^. Briefly, tissue samples were fixed using 4% buffered formalin and embedded in paraffin. Per patient one tissue core measuring 0.6 mm in diameter was extracted from a tumor-containing block, using a semi-automated custom-built tissue arrayer.

**Table 1:** Patient cohort.

Histological subtype		
Study cohort	858 (100.0%)	
Adenocarcinoma	470 (54.8%)	
Squamous cell carcinoma	235 (27.4%)	
Mesothelioma	50 (5.8%)	
Carcinoid	52 (6.1%)	
Large cell neuroendocrine carcinoma	16 (1.9%)	
Large cell carcinoma	7 (0.8%)	
Carcinosarcoma	2 (0.2%)	
Pleomorphic carcinoma	14 (1.6%)	
Adenosquamous carcinoma	8 (0.9%)	
Low-grade mucoepidermoid carcinoma	1 (0.1%)	
Lymphoepithelial carcinoma	1 (0.1%)	
SMARCA4-deficient undifferentiated tumor	2 (0.2%)	
Adenocarcinoma only		
Follow up	Months (mean)	15.2
	Censored (alive)	383 (88.9%)
	Failed (dead)	48 (11.1%)
Gender	Female	258 (55.8%)
	Male	204 (44.2%)
Stage	pT1	157 (36.0%)
	pT2	165 (37.8%)
	pT3	58 (13.3%)
	pT4	56 (12.8%)
Nodal stage	pN0	280 (68.6%)
	pN +	128 (31.4%)
Grade	1–2	69 (37.9%)
	3	113 (62.1%)
Resection margin	R0	420 (93.8%)
	R +	28 (6.2%)
Squamous cell carcinoma only		
Follow up	Months (mean)	14.9
	Censored (alive)	172 (83.1%)
	Failed (dead)	35 (16.9%)
Gender	Female	70 (30.3%)
	Male	161 (69.7%)
Stage	pT1	58 (25.5%)
	pT2	66 (29.1%)
	pT3	43 (18.9%)
	pT4	60 (26.4%)
Nodal stage	pN0	134 (59.8%)
	pN +	90 (40.2%)
Grade	1–2	35 (38.0%)
	3	57 (61.9%)
Resection margin	R0	204 (90.7%)
	R +	21 (9.3%)

Percent in the column “study cohort ” refers to the fraction of samples across each category. Because of cases with missing data, the numbers in the different categories do not always add up to the total number of cases.

### Immunohistochemistry (IHC)

Our IHC protocol has previously been described in detail^44^. Briefly, immunostaining was performed on freshly cut TMA-sections and experiment conducted on one day. Following deparaffinization with xylol and rehydration in a graded alcohol series, antigen retrieval was performed in an autoclave (121 °C) using Dako Target Retrieval Solution (Agilent Technologies, Santa Clara, CA, USA; #S2367) at pH 9 for 5 min. Using Dako REAL Peroxidase-Blocking Solution (Agilent Technologies, Santa Clara, CA, USA; #S2023) peroxidase activity was blocked for 10 min. The primary antibody binding E-cadherin (rabbit monoclonal, MSVA-035R, MS Validated Antibodies GmbH, Hamburg, Germany, #2140-035R-01, 1:150) was incubated for 60 min at 37 °C and visualization was performed using the Dako REAL EnVision Detection System Peroxidase/DAB + , Rabbit/Mouse kit (Agilent Technologies, Santa Clara, CA, USA; #K5007). Lastly, TMA-sections were counterstained using hemalaun. Analysis of tissue spots was performed on tumor tissue and membranous staining intensity (0, 1–3 +) as well as the percentage of positive tumor cells was recorded. To perform subsequent statistical analysis, staining results were grouped into negative, weak, moderate and strong. Tissues were considered negative if no staining was detectable and were considered weakly positive if ≤ 70% of tumor cells were stained with a 1 + staining intensity or if ≤ 30% of tumor cells were stained with a 2 + staining intensity. To be considered moderatley positive, tumor cells were stained either 1 + , > 70% or 2 + , 31–70% or 3 + , > 30%. To be considered strongly positive tumors stained either 2 + , > 70% or 3 + , > 30%. Non-interpretable samples demonstrated lack of unequivocal tumor cells or lack of entire tissue spots. This scoring system has previously been proposed by Simon et al. for the analysis of TMA studies^45^. It enables clear separation of negative cases and graded positive groups. Its routine use in numerous previous publications has demonstrated the ability to reliably reproduce known associations between marker staining intensity, clinicopathological features, and patient outcomes, supporting its suitability for prognostic analyses^41,44,46,47^. As several studies have demonstrated that involving multiple pathologists or non-pathologists to read the same TMA slides does not significantly impact the study outcome, all tissue spots were analyzed by a single experienced pathologist^48–51^.

### Statistics

All statistical analyses were conducted using JMP 17^®^ software (SAS Institute Inc., Cary, NC, USA). Associations between E-cadherin expression and tumor characteristics were evaluated using contingency tables and the chi-square (χ^2^) test. Survival probabilities were estimated using the Kaplan–Meier method, and differences between survival curves were assessed with the log-rank test.

## Results

### E-cadherin expression by tumor type

**Fig. 1:**
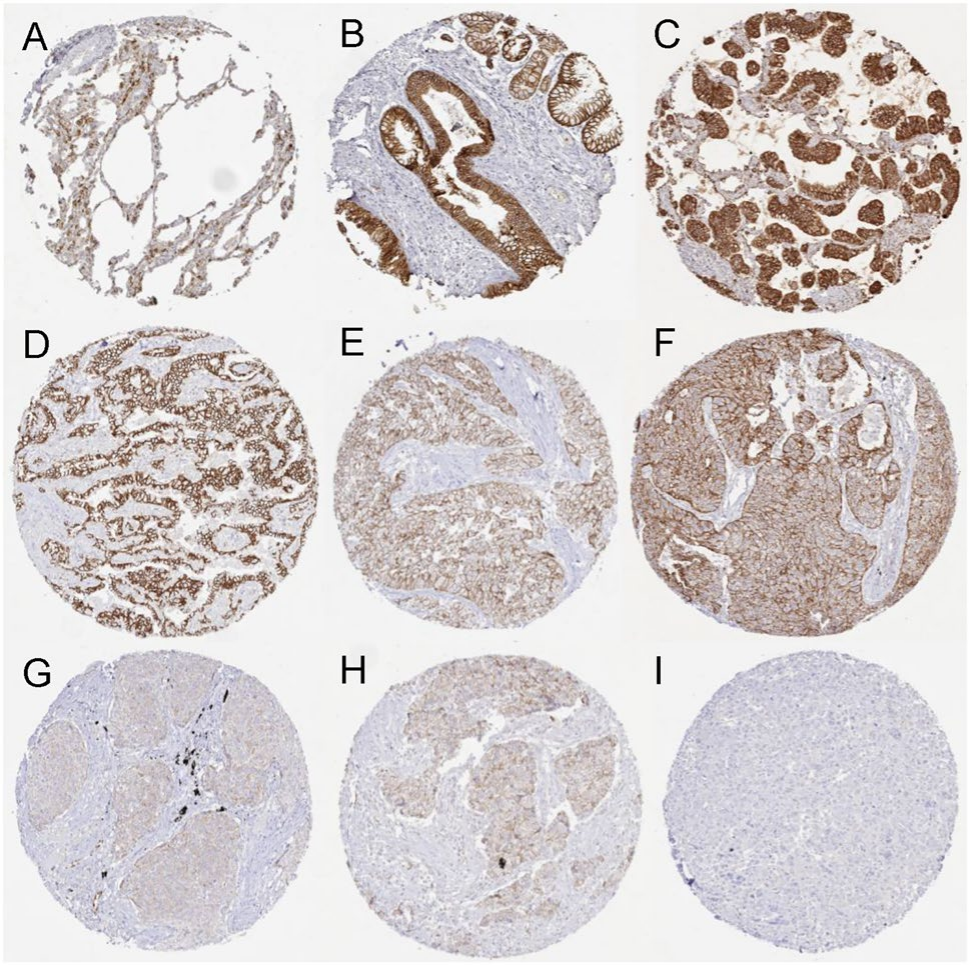
Membranous E-cadherin immunostaining in normal and neoplastic lung tissue. E -cadherin staining was moderate in the alveolar cells of the lung (**A**), strong in (non-basal) cells of the respiratory epithelium and the bronchial glands (**B**), strong in adenocarcinoma (**C**) and in squamous cell carcinoma (**D**), moderate in adenocarcinoma (**E**) and in squamous cell carcinoma (**F**), weak in an adenocarcinoma (**G**) and in squamous cell carcinoma (**H**), and negative in adenocarcinoma (**I**).

A total of 804 (93.8%) of 857 tumor samples were interpretable in our TMA analysis. Among normal tissues, E-cadherin immunostaining was predominantly membranous. It was considered moderate in alveolar cells of the lung and strong in respiratory epithelium and the bronchial glands (Fig. 1). In malignant tissues E-cadherin staining was always and predominantly membranous (Fig. 1). E-cadherin staining was seen in 779 (96.9%) of the 804 interpretable cancers, including 34 (4.2%) with weak, 161 (20.0%) with moderate, and 584 (72.6%) with strong positivity (Table 2). A loss of E-cadherin was observed in 25 (3.1%) of all NSCLC samples (Table 2). Representative images are shown in Fig. 1. E-cadherin staining was significantly more intense in AC (n = 438; weak 2.7%, moderate 8.7%, strong 87.4%) than in SCC (n = 233; weak 5.6%, moderate 31.3%, strong 63.1%), with ACs showing a higher rate of negative E-cadherin expression compared to SCCs (AC 1.1%, SCC 0.0%) (p < 0.0001) (Table 2). Representative images are shown in Fig. 1. Among the less common tumor entities, E-cadherin immunostaining was absent in 57.1% of 35 mesotheliomas, but retained in all cases of carcinoid (n = 52), large cell neuroendocrine carcinoma (n = 19), undifferentiated large cell carcinoma (n = 3), carcinosarcoma (n = 2), pleiomorphic carcinoma (n = 11), adenosquamous carcinoma (n = 8), mucoepidermoid carcinoma (n = 1), lymphoepithelial carcinoma (n = 1), and SMARCA4-deficient undifferentiated tumors (n = 1) (Table 2).

**Table 2:** E-cadherin immunostaining in lung cancer.

	n	E-cadherin immunostaining				P
		Negative (%)	Weak (%)	Moderate (%)	Strong (%)	
All lung cancers	806	3.1	4.2	20.2	72.5	
Adenocarcinoma	448	1.1	2.7	10.0	86.2	< 0.0001
Squamous cell carcinoma	223	0.0	5.8	30.0	64.1	
Mesothelioma	36	55.6	8.3	33.3	2.8	
Carcinoid	52	0.0	5.8	46.2	48.1	
Large neuroendocrine carcinoma	15	0.0	0.0	33.3	66.7	
Large cell carcinoma	7	0.0	42.9	28.6	28.6	
Carcinosarcoma	2	0.0	0.0	0.0	100.0	
Pleomorphic carcinoma	11	0.0	0.0	45.5	54.5	
Adenosquamous carcinoma	8	0.0	0.0	12.5	87.5	
Low-grade mucoepidermoid carcinoma	1	0.0	0.0	0.0	100.0	
Lymphoepithelial carcinoma	1	0.0	0.0	0.0	100.0	
SMARCA4-deficient undifferentiated tumor	2	0.0	0.0	100.0	0.0	

### Associations with clinicopathologic parameters

Reduced E-cadherin staining was significantly linked to advanced pT stage (p = 0.0265) and high grade (p = 0.0351) in ACs (Table 3). In SCC, low E-cadherin staining was associated with positive resection margins (p = 0.0029), but not with advanced pT stage, nodal metastasis or higher grade.

**Table 3:** E-cadherin and cancer phenotype.

		n	E-cadherin immunostaining				P
			Negative (%)	Weak (%)	Moderate (%)	Strong (%)	
Adenocarcinoma only	pT1	146	0.0	2.1	11.0	87.0	0.0127
	pT2	161	0.0	3.1	6.2	90.7	
	pT3	54	1.9	5.6	18.5	74.1	
	pT4	54	5.6	1.9	11.1	81.5	
	pN0	264	0.4	1.9	9.1	88.6	0.0483
	pN +	125	2.4	4.8	13.6	79.2	
	G1-2	64	0.0	0.0	0.0	100.0	0.0351
	G3	109	0.9	0.9	6.4	91.7	
	R0	399	1.0	2.3	9.8	87.0	0.0436
	R +	27	3.7	11.1	18.5	66.7	
Squamous cell carcinoma only	pT1	54	0.0	3.7	18.5	77.8	0.1477
	pT2	65	0.0	3.1	30.8	66.2	
	pT3	38	0.0	5.3	34.2	60.5	
	pT4	58	0.0	10.3	36.2	53.4	
	pN0	124	0.0	4.8	29.8	65.3	0.8076
	pN +	88	0.0	6.8	30.7	62.5	
	G1-2	33	0.0	3.0	15.2	81.8	0.9831
	G3	53	0.0	3.8	15.1	81.1	
	Any G	86	0.0	3.5	15.1	81.4	
	R0	193	0.0	4.7	28.0	67.4	0.0147
	R +	20	0.0	15.0	50.0	35.0	

*pT* pathological tumor stage, *G* Grade, *pN* pathological lymph node status, *R* resection status.

### Survival analysis

**Fig. 2:**
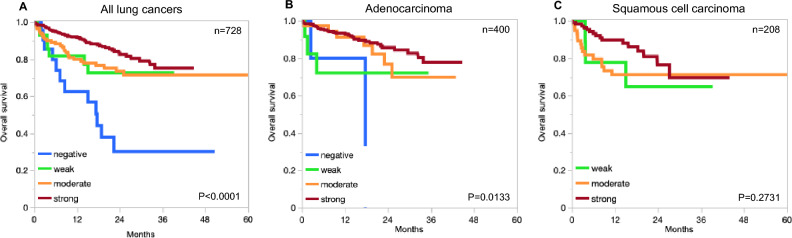
E-cadherin and prognosis in lung cancer. The panels show the impact of E-cadherin staining levels on overall survival in all lung cancers (**A**), AC (**B**), and SCC (**C**).

Univariate outcome analysis revealed a significant association of E-cadherin expression with overall survival in all lung tumors (p < 0.0001) and in ACs (p = 0.0133), if tumor groups with negative, weak, moderate, and strong staining were compared (Fig. 2).

## Discussion

The successful analysis of more than 800 lung cancers revealed a high rate of E-cadherin positive ACs (98.9%) and SCCs (100%). More than 100 previous studies have investigated E-cadherin expression by IHC in lung cancer in cohorts of n = 1–635 NSCLCs and reported highly variable positivity rates ranging from 19.4 to 100% (average 49.9%) in AC^24,52–54^ and from 7.0–100% (average 50.1%) in SCC (Supplementary Fig. 1)^34,52^. Such variability might be attributed to methodological heterogeneity, including variations in IHC protocols, antibody sensitivity, thresholds defining positivity, and clinico-pathological differences of patient cohorts. Our positivity rates for E-cadherin are at the upper end of the previously reported range of data. It is of note, that our assay had earlier been validated according to the guidelines of the International Working Group for Antibody Validation (IWGAV)^41,55^ by comparison of IHC data obtained by our assay on 76 different normal tissue types with RNA expression data^56–58^ and IHC results obtained by an independent second antibody. This approach enabled a comprehensive assessment of nearly all proteins and their posttranslational modifications for potential cross-reactivity.

That mesothelioma exhibited the lowest rate of E-cadherin positive immunostaining in our cohort is consistent with data from prior reports^53,54,59–64^. Although the difference in E-cadherin expression between ACs and SCCs reached statistical significance, loss of expression occurred only slightly more frequently in ACs than in SCCs. Given this subtle difference, it is not surprising that findings with respect to differences between ACs and SCCs were highly inconsistent across previous studies (Supplementary Figure 1)^12,14–18,20,21,23,25–28,34,40,52,65–78^.

Our data demonstrate a significant association between reduced E-cadherin expression and poor prognosis as well as an unfavorable tumor phenotype in pulmonary ACs (Fig. 2, Table 3). While numerous studies have described a prognostic role of E-cadherin expression levels in combined series of different subtypes of NSCLCs, only few have evaluated ACs and SCCs as distinct entities. In line with our findings, 4 previous studies evaluating the prognostic impact of E-cadherin expression in cohorts of 31–117 ACs have found associations between low E-cadherin and unfavorable clinic-pathological tumor features^24,29–31^ as well as poor patient prognosis^29–31^ while 3 other studies failed to establish a significant correlation between E-cadherin expression and those parameters^22,37,38^. Considering the rigorous validation of our assay and the highly standardized experimental conditions applied in this study, our results provide a technically refined large-scale confirmation of earlier observations, strengthening the evidence for prognostic associations of E-cadherin expression in NSCLC while simultaneously offering a comprehensive overview of its expression across different thoracic cancers.

That the reduced expression of E-cadherin was associated with impaired patient survival in pulmonary ACs aligns with previously published data by us and others, which described associations between reduced or absent E-cadherin expression and poor prognosis in many different cancer entities^79^, and demonstrated a particularly high frequency of E-cadherin loss in highly lethal cancers with dedifferentiated morphology^41,53,54,59–63,80–83^. Different aspects of cancer cell biology may explain how reduced E-cadherin expression contributes to an aggressive tumor phenotype and subsequent poor patient prognosis^79^. A reduction in membranous E-cadherin, a central step in EMT^84^, disrupts epithelial cell–cell adhesion by weakening adherens junctions, thereby promoting tumor cell migration and invasion^85^. In addition, E-cadherin exerts tumor-suppressive functions by anchoring β-catenin at the cell membrane and by interacting with growth factor receptors such as EGFR, IGF-1R, and c-Met, thereby limiting oncogenic Wnt signaling and growth-promoting pathways^79,86–89^.

That the prognostic role of reduced E-cadherin expression was more limited in SCCs is expected given that our highly sensitive IHC protocol only resulted in a low rate of negative or low positive cases in our cohort (Fig. 2, Tables 2 and 3). Although the small size of our subgroup with low E-cadherin expression did not allow for a powered statistical discrimination, this finding is consistent with most previous studies analyzing the prognostic role of E-cadherin in pulmonary SCCs that had failed to find a prognostic role of E-cadherin loss^24,31–33,39,90^.

Several molecular mechanisms have been implicated in reduced membranous E-cadherin expression in NSCLC. Genetic alterations of the CDH1 gene, including inactivating mutations (~ 0.2%) and deletions at chromosome 16q22.1 (~ 0.12%) are rare, while epigenetic regulation through hypermethylation has been reported at a rate of up to 34%^91–94^. Transcriptional repression by EMT-associated factors including ZEB1/2^95^, TWIST1^96^ and SNAIL2^97^, have also been described. Additional mechanisms include posttranscriptional regulation through microRNAs^98^, post translational modifications^99^, proteolytic cleavage of E-cadherin^100^, and disruption of the E-cadherin/β-catenin complex through altered p120- or β-catenin function^101,102^.

Although loss of E-cadherin expression was rare in our NSCLC cohort and restricted to ACs, therapeutic strategies aimed at restoring E-cadherin function are currently being explored^103^. These approaches include targeting CDH1 promoter hypermethylation with demethylating agents^104–106^, inhibition of TGF-β or AXL signaling to counteract EMT^107,108^, blocking ADAM proteases involved in E-cadherin cleavage^109^, modulation of post-transcriptional regulation through miRNAs^110^, and the use of monoclonal antibodies to stabilize the E-cadherin–catenin complex^111,112^. Several of these strategies are currently under evaluation in early-phase clinical trials in NSCLC^113–116^.

In conclusion, this study demonstrates that E-cadherin expression is reduced in a small fraction of NSCLCs, that a loss of E-Cadherin expression is more common in ACs than in SCCs, and that a disturbed E-Cadherin expression is associated with poor prognosis in ACs. Should targeted therapy become readily available in the future, AC of the lung could potentially represent an indication for such treatment in a fraction of cases.

## Supplementary Information

Supplementary Information.

## Data Availability

All data generated or analyzed during this study are included in this published article.

## Abbreviations

NSCLC: Non-small lung carcinoma
AC: Adenocarcinoma
SCC: Squamous cell carcinoma
EMT: Epithelial to mesenchymal transition
IHC: Immunohistochemistry
TMA: Tissue microarray
IWGAV: International Working Group of Antibody Validation
