# Supplementary material for: Low E-cadherin expression is associated with poor prognosis in pulmonal adenocarcinoma
Source: Sci Rep. 2026 Mar 30;16:10663. doi: 10.1038/s41598-026-45409-0 (PMC13039912; doi:10.1038/s41598-026-45409-0)
Supplement: Supplementary file 1 — Supplementary Information. [file 41598_2026_45409_MOESM1_ESM.pptx]

## Slide 1
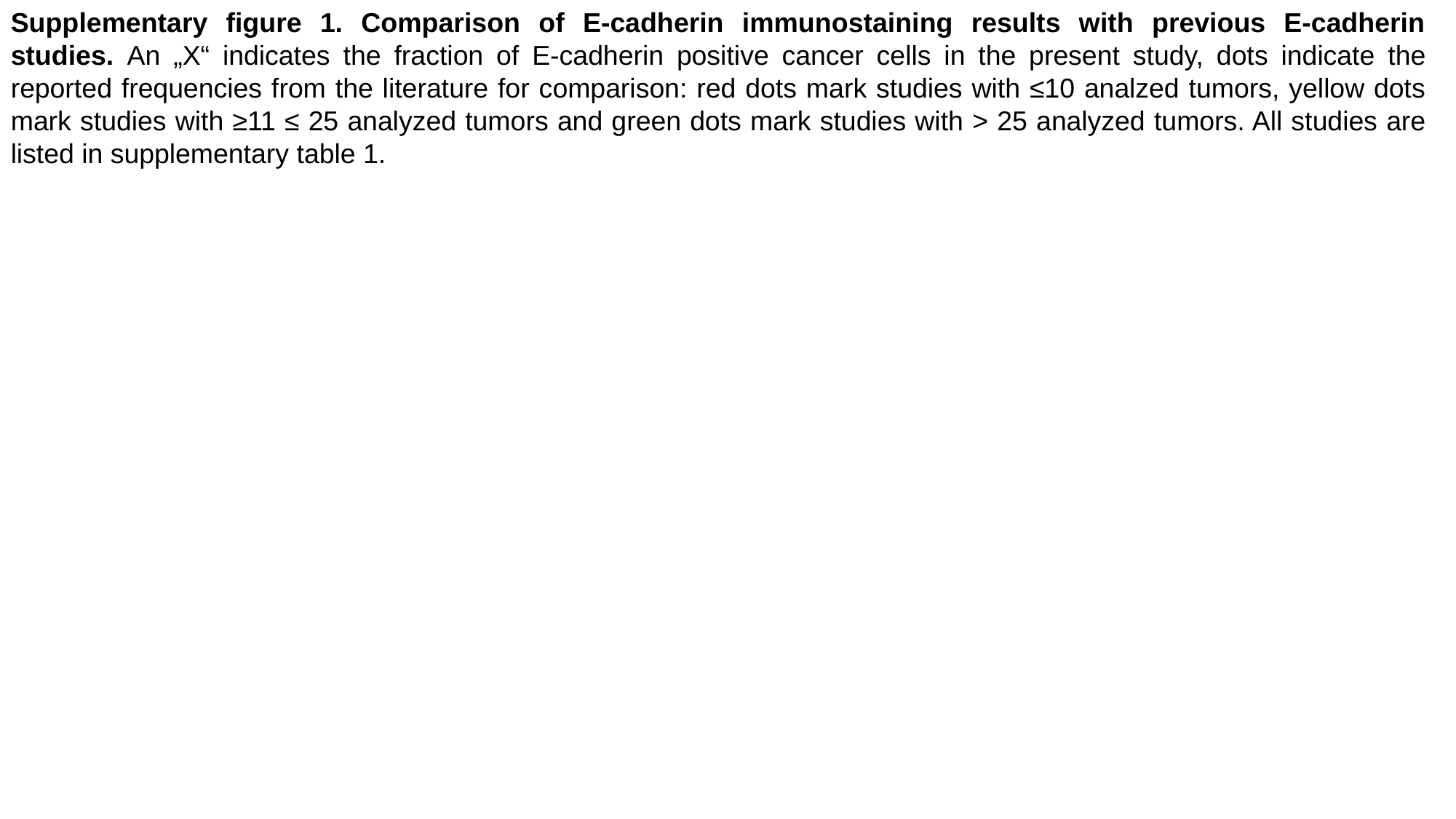

Supplementary figure 1. Comparison of E-cadherin immunostaining results with previous E-cadherin studies. An „X“ indicates the fraction of E-cadherin positive cancer cells in the present study, dots indicate the reported frequencies from the literature for comparison: red dots mark studies with ≤10 analzed tumors, yellow dots mark studies with ≥11 ≤ 25 analyzed tumors and green dots mark studies with > 25 analyzed tumors. All studies are listed in supplementary table 1.
